# [Retracted] Role of miR-146a in human chondrocyte apoptosis in response to mechanical pressure injury *in vitro*

**DOI:** 10.3892/ijmm.2026.5826

**Published:** 2026-04-07

**Authors:** Lei Jin, Jian Zhao, Wensen Jing, Shiju Yan, Xin Wang, Chun Xiao, Baoan Ma

Int J Mol Med 34: 451-463, 2014; DOI: 10.3892/ijmm.2014.1808

Following the publication of the above article, a concerned reader drew to the Editor's attention that certain of the Smad4, VEGF and GAPDH protein bands featured in Figs. 3D, 5C and F, 8D and 10B appeared to be strikingly similar, suggesting that the data featured in the affected figure parts had been erroneously assembled. After having conducted an internal investigation of the data in this paper in the Editorial Office, the findings of the reader were found to be largely confirmed; in particular, it was noted that the western blots featured in Figs. 5C, 5F and 10B were strikingly similar, even though the orientation of the bands differed in some of the gel slices, such that data which were intended to show the results of differently performed experiments had apparently all been derived from the same original source.

Given the large number of identifications of overlapping and/or re-used western blot data in this paper, the Editor of *International Journal of Molecular Medicine* has determined that this paper should be retracted from the Journal on the grounds of an overall lack of confidence in the data. The authors were asked for an explanation to account for these concerns, but the Editorial Office did not receive a satisfactory reply. The Editor sincerely apologizes to the readership for any incovenience caused, and we thank the reader for drawing this matter to our attention.

